# Rapid Spatial Learning Controls Instinctive Defensive Behavior in Mice

**DOI:** 10.1016/j.cub.2017.03.031

**Published:** 2017-05-08

**Authors:** Ruben Vale, Dominic A. Evans, Tiago Branco

**Affiliations:** 1 MRC Laboratory of Molecular Biology, Francis Crick Avenue, Cambridge CB2 0QH, UK; 2 UCL Sainsbury Wellcome Centre for Neural Circuits and Behaviour, Howland Street, London W1T 4JG, UK

**Keywords:** innate behavior, defensive behavior, escape, freezing, mouse, spatial learning, spatial memory, shelter

## Abstract

Instinctive defensive behaviors are essential for animal survival. Across the animal kingdom, there are sensory stimuli that innately represent threat and trigger stereotyped behaviors such as escape or freezing [1, 2, 3, 4]. While innate behaviors are considered to be hard-wired stimulus-responses [5], they act within dynamic environments, and factors such as the properties of the threat [6, 7, 8, 9] and its perceived intensity [1, 10, 11], access to food sources [12, 13, 14], and expectations from past experience [15, 16] have been shown to influence defensive behaviors, suggesting that their expression can be modulated. However, despite recent work [2, 4, 17, 18, 19, 20, 21], little is known about how flexible mouse innate defensive behaviors are and how quickly they can be modified by experience. To address this, we have investigated the dependence of escape behavior on learned knowledge about the spatial environment and how the behavior is updated when the environment changes acutely. Using behavioral assays with innately threatening visual and auditory stimuli, we show that the primary goal of escape in mice is to reach a previously memorized shelter location. Memory of the escape target can be formed in a single shelter visit lasting less than 20 s, and changes in the spatial environment lead to a rapid update of the defensive action, including changing the defensive strategy from escape to freezing. Our results show that although there are innate links between specific sensory features and defensive behavior, instinctive defensive actions are surprisingly flexible and can be rapidly updated by experience to adapt to changing spatial environments.

## Highlights

• Mice instinctively escape to a previously memorized shelter location
• Orientation and navigation to the shelter does not require shelter-related cues
• Memory of the shelter location is formed in a single trial
• Changes in the spatial environment lead to rapid updates of defensive actions

## Teaser

Vale et al. show that mice exposed to innately aversive stimuli instinctively escape to a shelter location that was learned during exploration of the environment. Memory of shelter existence and location is formed in a single trial and can be rapidly updated, allowing mice to quickly adapt defensive actions to changes in the spatial environment.

## Results

### Escape Behavior Is a Goal-Directed Action to Reach Safety

**Figure 1:**
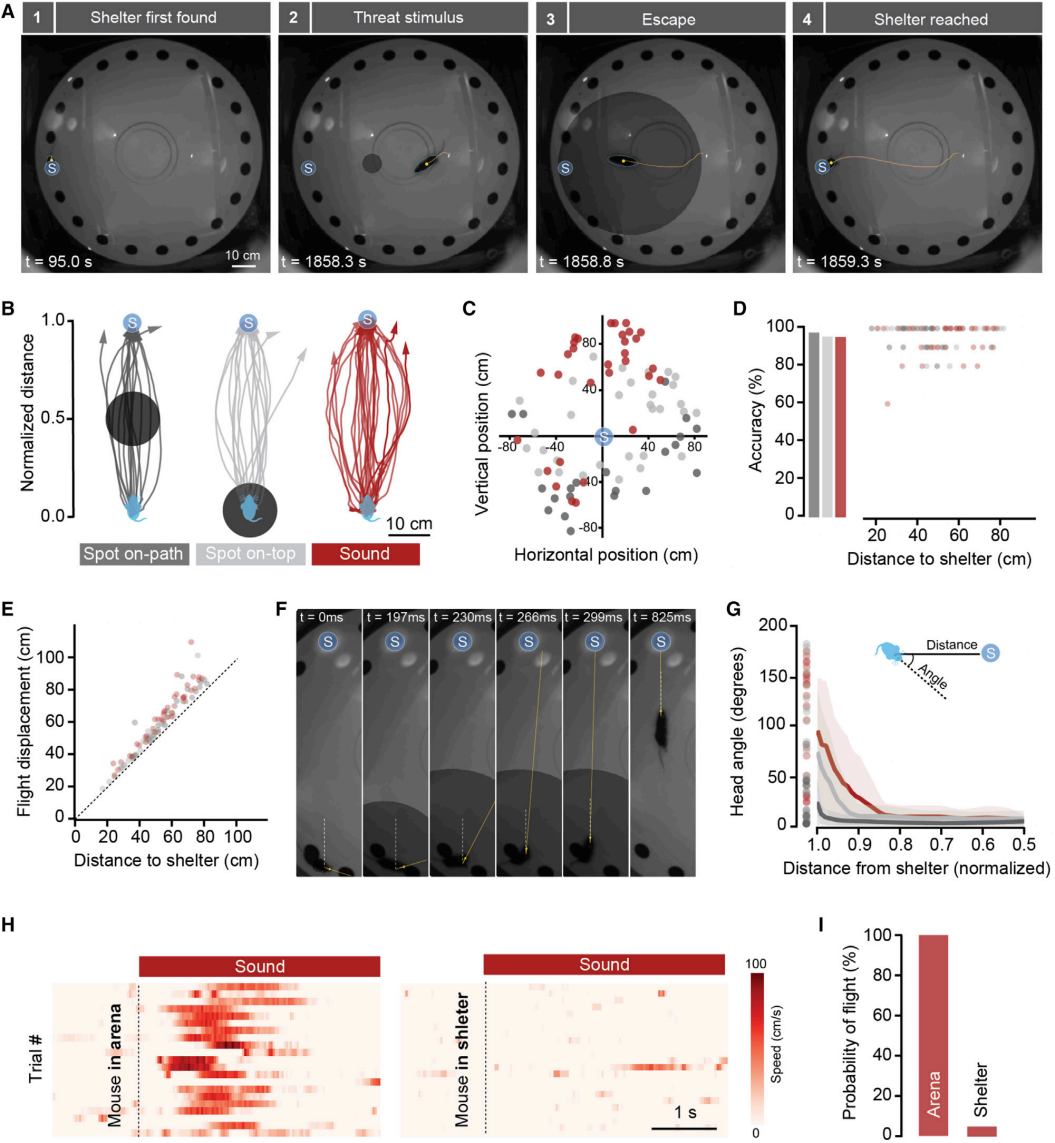
Escape Behavior Is a Goal-Directed Action to Reach Safety (A) Video frames from one trial showing escape to a previously explored shelter after stimulation with an expanding spot projected from above, between the mouse and the shelter location (on-path). Yellow lines indicate the mouse’s trajectory during the preceding 2 s. (B) Example trajectories from several mice, recorded between stimulus onset and the end of flight, showing that flight path and target are independent of stimulus position or quality (number of animals = 10 on-path, 16 on-top, and 15 sound). (C) Initial position of mice in all trials plotted in relation to the shelter location. (D) Accuracy of reaching the shelter during escape. Bars show average accuracy and circles are individual accuracy data points as function of distance to the shelter. (E) Total displacement during escape for 100% accurate flights plotted against linear distance to the shelter. (F) Video frames from one trial during initiation of escape from an expanding spot on-top, highlighting the initial head rotation preceding the initiation of running. The yellow line indicates head direction, and the dashed white line is the reference line between the current mouse position and the shelter. (G) Head angles measured between the white and yellow lines illustrated in (F) for 100% accurate flights, showing that the head is pointing toward the position of the shelter before the distance to the shelter is covered. Circles indicate the initial angles for different trials, lines indicate average head rotation profile, and shaded areas indicate the SD (n = 59 trials from 38 animals). (H) Raster plots showing speed profile of trials in several mice stimulated with sound when exploring the arena (left) or when the same mice were inside an over-ground shelter (right). (I) The probability of flight is dramatically reduced when animals are already inside a shelter. For all relevant panels, the blue circle with “S” identifies the shelter location and dark gray, light gray, and red indicate data from stimulation with spot on-path, spot on-top, and sound, respectively. See also Figure S1 and Movie S1.

When escaping from imminent threat, animals have two general options: to move away from the threat or to move toward safety. These two behaviors have different consequences and are fundamentally distinct in the computations they require. Moving away from threat can be implemented as a simple reaction to the stimulus [22], but it has the drawback that it might not be the most adaptive solution, if it increases detectability or the animal moves into a position from which it cannot escape [3, 23]. On the other hand, moving toward a safe place has better long-term value but requires more complex computations that might take valuable time, such as evaluating shelter locations and available escape routes. To test which strategy is preferentially used by mice exposed to innately aversive threats, we placed naive animals in a Barnes maze, which is a circular arena with 20 identical holes that are all covered except for one that leads to an underground shelter [24] (Figure 1A). After a short habituation period (7 min) during which mice spontaneously found the shelter location, we exposed them to overhead dark expanding spots, previously shown to be innately aversive [4], delivered either between the mouse and the shelter (on-path) or directly above the mouse (on-top). Both stimuli elicited fast escape to the shelter with short reaction times (202 ± 16 ms; n = 51 responses from 26 animals; Figure 1B; Movie S1) independently of the initial location of the mouse (Figure 1C). Surprisingly, we found no relationship between the stimulus position and the evoked escape trajectories, which were all directed to the shelter, even when the stimulus was between the mouse and the shelter, requiring the mouse to run toward the aversive stimulus in order to reach safety (Figure 1B). In contrast with trajectories during foraging, flight trajectories were very close to a straight line and were not different between the two stimulus conditions (mean linearity ratio: on-path, 106% ± 1%; on-top, 109% ± 2%; foraging, 209% ± 30%; p = 0.27, t test between on-path and on-top; p < 0.0001, t test between flights and foraging), as well as highly accurate (mean accuracy: on-path, 89% ± 5%; on-top, 97% ± 1%; p = 0.32, t test between on-path and on-top), despite the lack of any long-term training (Figures 1D and 1E). In addition, the first body movement after the onset of the stimulus was head orientation toward the shelter location. This orienting behavior was independent of the initial angle between the head direction and the shelter, which was reduced to less than 10° before the mouse covered the first 10% of the distance to shelter and thus preceded the onset of full flight (Figures 1F, 1G, and S1). Remarkably, in 91.5% of the trials, mice rotated their head toward the side of the narrower angle, indicating an awareness of the flight target before the onset of head turning. Similar behavior was observed in response to overhead ultrasonic sweeps [25], which represent a more spatially diffuse threat (Figures 1B–1G; see the Experimental Procedures) and further support the independence of the behavior from threat localization in this environment.

These data suggest that the goal of the escape behavior is to reach safety. To further test this hypothesis, we reasoned that presentation of the threat while the animal is in the shelter should not cause escape behavior. Indeed, auditory stimuli delivered both in the Barnes maze and in a modified version with a surface shelter did not cause escape behavior, despite the sound pressure level inside the shelter being within 2dB of the arena outside (escape probability = 100% outside versus 6% inside; p < 0.001, t test between the two conditions; n = 76 responses from 11 animals; Figures 1H and 1I), indicating that the perception of safety can veto escape from innately aversive threats. These results show that instinctive escape behavior in the mouse is not a simple stimulus reaction, but a generic action in response to threat with the goal of reaching a safe area, the location of which is computed before the onset of the escape.

### Memory of Shelter Location Guides Defensive Flight

**Figure 2:**
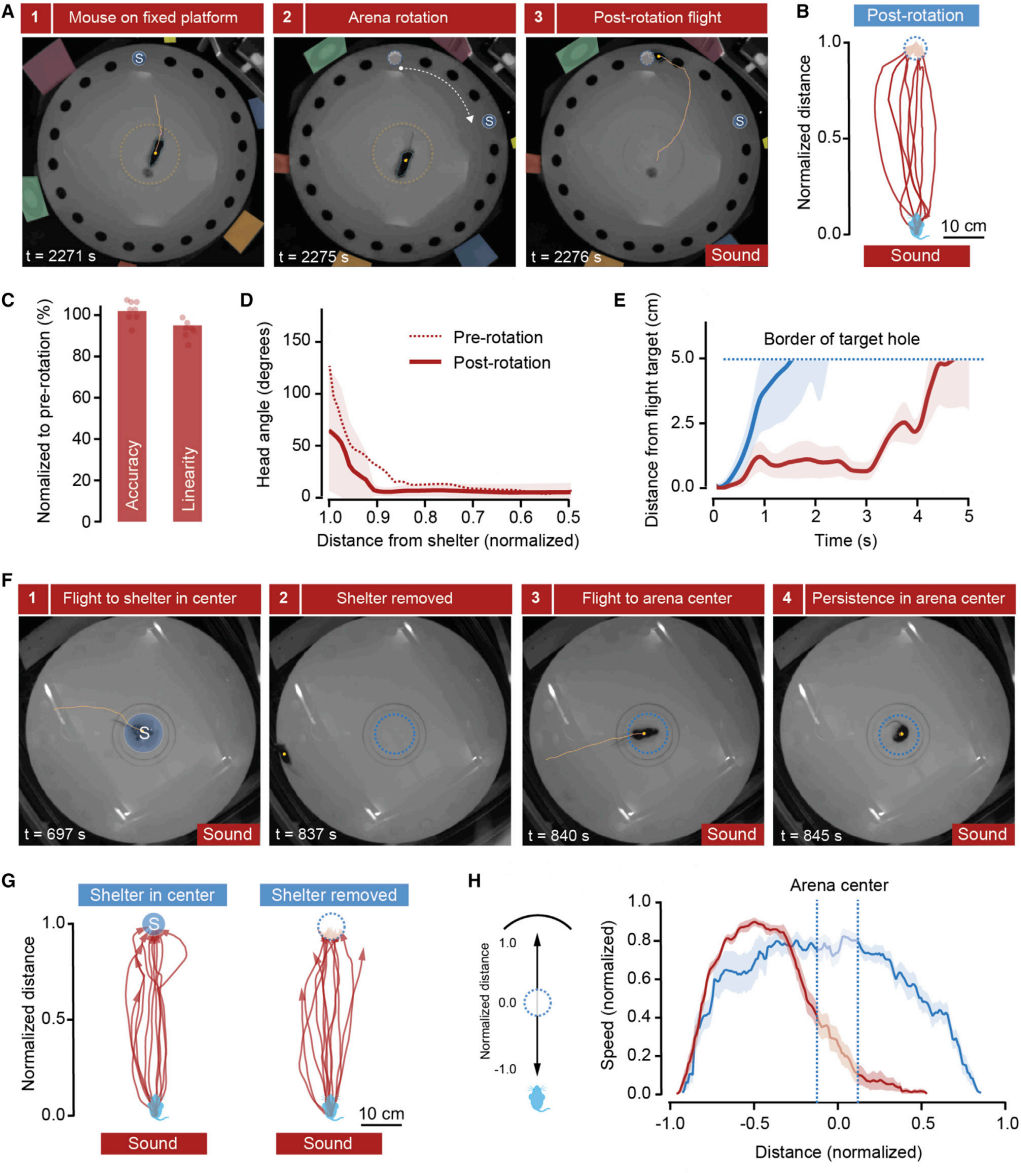
Memory of Shelter Location Guides Defensive Flight (A) Video frames from one trial showing escape from aversive sound immediately after the outside of the arena had been rotated, together with local cues (panels on the outside, color-coded for clarity). The dashed yellow line marks the diameter of the fixed platform, and the dashed blue circle shows shelter location before rotation. (B) Trajectories from different mice after arena rotation, showing escape toward the previous shelter location (dashed blue circle). (C) Escape behavior is not significantly changed by arena rotation (accuracy = 102% ± 1%, linearity = 96% ± 2% of control). Reaction time is also not affected (93% ± 14%). p > 0.1 for all comparisons, paired t test between pre- and post-rotation; n = 8 animals. (D) Head rotation profile during escape initiation is not affected by arena rotation (p = 0.39, paired t test between pre- and post-rotation for distance at 10°). Post-rotation angles are measured between the mouse position and the shelter position before rotation. The shaded area indicates the SD. (E) Plot showing when the mouse leaves the initial target hole area after the flight. Red indicates flights after rotation, and blue indicates flights in control conditions where the shelter target was missed. The shaded area indicates the SEM. (F) Video frames from one trial showing sound-evoked flight to a shelter in the center of the arena and persistence of escape to the arena center after the shelter has been removed. (G) Escape trajectories for different mice before (left) and after (right) a shelter in the arena center was removed. (H) Speed profile for escape responses when the shelter is in the periphery (blue; from the same dataset shown in Figure 1) and after the shelter has been removed from the arena center. See also Figure S2 and Movie S2.

We next investigated the strategies mice use to determine shelter location. Previous work has shown that foraging rodents can navigate using a variety of strategies [26, 27], including retrieval of a cognitive spatial map [28], relying on prominent external landmarks [26], and integrating self-motion cues over time (path integration [29, 30]). Here, we tested whether spatial landmarks in the local surroundings of the shelter are used to guide escape and whether flight termination is signaled by the safety conferred by arriving inside the shelter. We performed two complementary experiments. First, we placed animals in a modified Barnes maze in which the center was fixed and the periphery could be automatically rotated, together with a set of olfactory and visual local cues that have been shown to guide navigation in mice [31]. Escape responses to the shelter were first elicited with sound stimuli, after which the peripheral ring of the arena was rotated by a random angle when mice were in the center (range = 36°–90°, mean = 56°; corresponding to two to five holes, mean = 3.1) and the sound stimulus was delivered again (Figure 2A; Movie S2). All mice invariably ran toward the previous shelter location, with accuracy, trajectory linearity, reaction times, and head orientation profile that were not different from those of pre-rotation flights (Figures 2B–2D). Moreover, mice stayed in the vicinity of the pre-rotation location for 4.6 ± 0.2 s, which is 2.5 times longer than the time mice spent in the wrong location during missed flights in control conditions (Figure 2E; p < 0.001, t test for time in the wrong location between control and post-rotation), further indicating goal directedness toward this location. These data suggest that landmarks proximal to the shelter are not required for the computation of shelter location, and this is further supported by threat presentation in complete darkness, which evokes perfectly accurate escape responses (Figures S2A and S2B; Movie S2). Next, we placed a shelter in the center of the arena, to which mice fled reliably when exposed to auditory stimulation, and then removed the shelter and repeated the auditory stimulation. Remarkably, this resulted in flights that stopped in the arena center (Figures 2F–2H; Movie S2) and were followed by persistence in this location, which is normally aversive to mice (Figure S2C), sometimes up to 15 s (mean = 2.5 ± 1.1 s). Together with the previous experiment, these results show that mice escape toward a previously memorized shelter location and that flight termination is signaled by having reached the stored target location and does not require reaching safety.

### Shelter Location Memory Is Formed Rapidly and Supports Fast Updates of Defensive Actions

**Figure 3:**
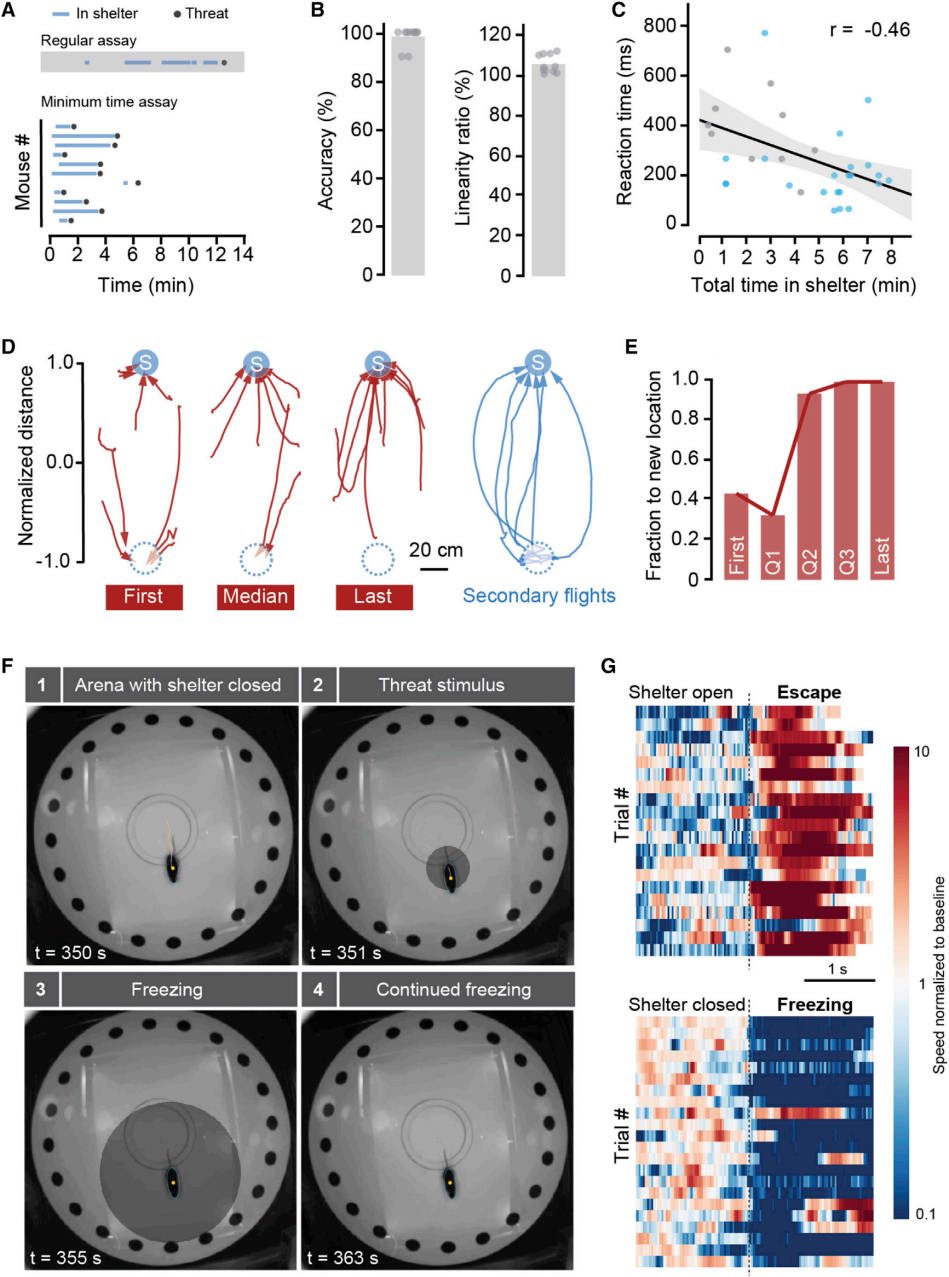
Shelter Location Memory Is Formed Rapidly and Supports Fast Updates of Defensive Actions (A) Raster plot showing periods of time inside the shelter from the onset of arena exploration and threat stimulus presentation. An example raster from a regular assay for comparison (as shown in Figure 1) with multiple entries in the shelter during the exploration phase is shown at the top. (B) Average (bars) and data points (circles) for accuracy and linearity of escape after shelter single visits. (C) Time to initiate escape is negatively correlated with the total amount of time spent in the shelter before stimulation. Gray circles are data from the minimum time assay, and blue circles are data from the regular assay. The black line is a regression line fit to all data points, and the shaded area is 95% confidence interval for the regression. (D) Escape trajectories after the original shelter has been closed (dashed blue circle) and a new one open in a different position (blue circle with “S”) for the first and last trials (left and right red, respectively) and the median trial (center red). Trajectories in blue (right) are for secondary flights, which immediately follow escapes to the original location. (E) Evolution of escape behavior after shelter location has been moved, as in (D), showing the fraction of flights across all mice that reach the new shelter location, for the first, three quartiles (Q1–Q3), and last trials. (F) Video frames from one mouse in an arena with the shelter closed, showing freezing behavior in response to a slowly expanding spot projected on top. (G) Raster plots showing speed profiles upon threat stimulation before (bottom) and after the shelter hole has been opened (top) for slowly expanding spots. Trials have been aligned by reaction time (dashed line). See also Figure S3 and Movie S3.

If mice rely on memory of the shelter location to reach it, how is this memory formed? To determine this, we removed the fixed habituation period and exposed animals to threat immediately after they visited the shelter for the first time. Even though animals were inside the shelter for as little as 18 s (range = 18–270 s; n = 12 animals; Figure 3A), this was enough to support shelter-directed escape responses that were indistinguishable from those of the control condition (Figure 3B; p = 0.79 for accuracy and p = 0.78 for linearity, t test against control). This shows that memory of shelter location is formed by a very fast single-trial learning process. Interestingly, there was a significant negative correlation between the total time spent in shelter and the reaction time (Pearson’s r = −0.46; p = 0.007), suggesting that computation of the escape vector might depend on the strength of the shelter location memory (Figure 3C).

We next investigated how shelter place memory supports updates of defensive actions when the environment changes by performing two sets of experiments. First, we elicited one flight with the sound stimulus in control conditions, after which we changed the location of the shelter to the opposite hole (see the Supplemental Experimental Procedures). We then waited until animals spontaneously visited the new shelter (mean time = 33.1 s; range= 4–82 s), after which we ran several trials of sound stimulation. We found that animals escaped to the new shelter location in less than two trials (mean = 1.8 ± 0.3 trials), with four out of nine mice escaping to the new location on the first trial. Some animals still escaped to the old location after having fled to the new one on a previous trial, but after four trials (mean value; over a period of 10.5 ± 6.8 min), nine out of nine animals escaped repeatedly to the new location (Figures 3D and 3E). Importantly, escapes to the old location were immediately followed by secondary straight flights to the new location (including four out of five first trial escapes to the old location; Figure 3D; Movie S3), suggesting that despite reaching the wrong target, mice already held the memory of the new shelter location. This shows that the new shelter location can be stored in a single trial and that safety devaluation of the old location supports a permanent update of the escape target after a small number of trials. In the second set of experiments, we closed the shelter hole, and after 7 min of exploration, during which animals always visited the closed shelter location, presentation of the visual stimulus directly above the mouse did not elicit escape, but instead caused freezing for the duration of the stimulus (freezing probability = 71.4%; mean freezing time = 629.9 ± 100.0 ms; flight probability = 10.7%; Figure S3), including long-lasting freezing for slowly expanding spots, sometimes lasting a long as 50 s (freezing probability = 95.2%; mean freezing time = 7.9 ± 2.7 s; flight probability = 4.8%; Figures 3F and 3G; Movie S3). This change in defensive strategy was completely reversible, as stimulus presentation 5 min after re-opening of the shelter hole once again produced robust shelter-directed flights (Figures 3G and S3). These data show that instinctive defensive escape is conditional on the knowledge of an existing shelter location and that in the absence of a memory of shelter location, mice switch their defensive strategy to freezing.

## Discussion

We have shown that instinctive defensive actions depend on rapidly learned information about the spatial environment and that the expectation of safety drives escape behavior to a learned shelter location, whereas its absence promotes defensive freezing. Our results support the idea that computations other than threat detection play an important role in the initiation of defensive behavior [32]. In our assay, there are at least two computational steps that precede defensive action: evaluation of whether shelter is available and, if so, determination of its location. The first is used to choose between fleeing or freezing, and the second is used to compute an escape vector from the current position to the shelter location, which we demonstrate to happen before flight initiation. Importantly, we show that information about the availability and location of the shelter is stored as a memory, which suggests that mice use spatial representations to coordinate instinctive defensive behaviors. This is in agreement with results from experiments in gerbils suggesting that spatial maps might be used to optimize escape routes [33]. In our experiments, the same visual stimulus could elicit both escape and freezing depending on the spatial configuration of the arena, and thus although it is possible that different defensive behaviors might be mediated by distinct visual pathways, as previously suggested [2], our results are compatible with a more general model in which sensory stimuli are incorporated into higher-order information streams to make the choice between freezing and fleeing from threat.

Previous studies on foraging rodents have shown that spatial navigation can be accomplished using both landmark information and self-motion cues and that when both are present, the most reliable information is used [29, 34, 35]. For example, homing hamsters will follow local cues that have been rotated, but only up to a certain angle, after which they switch strategies and perform path integration [34]. In our experiments, rotation of local landmarks did not change the accuracy of escape behavior, suggesting that self-motion cues might play an important role when fleeing from threats. Although we cannot rule out that landmarks outside our experimental control contribute to navigation, path integration is particularly well suited to compute the current position as a vector from a home base [36] and could be the preferred strategy during escape. This strategy might have the advantage that animals do not need to scan the environment for local cues that signal the shelter, which could take a significant amount of time and might thus shorten computation times. Interestingly, mice stop at the learned shelter location when the shelter is absent, even if the location is the arena center, suggesting that shelter cues and the safety conferred by the shelter are not processed during the escape response and are not necessary to terminate flight. A key finding of this study is that learning the shelter location is a very fast process requiring only a single visit and that flight accuracy is extremely high from the first escape trial. This contrasts with previous experiments using Barnes mazes, where the accuracy to find the shelter increases slowly over multiple trials across several days [31, 37]. An important difference is that in our experiments, threats were presented after mice moved away from the shelter voluntarily instead of being placed in the maze center by the experimenter [31, 37], further supporting the idea that path integration might be the dominant navigation strategy during escape.

A key consequence of rapid spatial learning is that it greatly increases the flexibility of escape behavior. We have shown that a single, short-lived visit to a shelter is sufficient to support accurate escape behavior and that changes in the environment are incorporated into action selection within minutes, suggesting that mice have very rapid mechanisms for risk assessment [38, 39]. Importantly, when we devaluated the outcome of the flight by moving the shelter to a new location, mice updated the flight goal within a few trials, indicating that the expected outcome of the defensive action might be taken into account and that instinctive escape could be considered within a model-based behavior framework [40]. In conclusion, although instinctive defensive behaviors rely on innate stimulus-response associations, their computation takes into account internal models of the world that are rapidly updated, and we suggest that they are a powerful model for investigating the neural basis of motivated action selection.

## Experimental Procedures

### Animals and Behavioral Procedures

All experiments were performed under the UK Animals (Scientific Procedures) Act of 1986 (PPL 70/7652) following local ethical approval. Male C57BL/6J mice were used for experiments at 6–12 weeks old and were tested during the light phase of the light cycle. The main behavioral arena used was a modified Barnes maze [24], consisting of a white acrylic circular platform 92 cm in diameter with 20 equidistant circular holes. The central area of the arena was a fixed circular platform, and the periphery was mounted on a frame that allowed rotation over 360°. The maze was surrounded by visual cues, and bedding from the home cage of the mouse being tested was placed inside the shelter. Experiments were recorded at 30–50 frames per second with a near-infrared camera. Unless otherwise noted, animals were given a 7 min acclimation period and an additional 5 min if they did not visit the shelter at least once. If the shelter was not found in this period, the experiment was terminated.

### Auditory and Visual stimulation

The auditory stimulus consisted of a train of three frequency modulated upsweeps from 17 to 20 kHz over 3 s [25], lasting 9 s in total, at a sound pressure level of 73–78 dB as measured at the arena floor. Visual stimuli were backprojected on to a screen positioned 64 cm above the arena and consisted of an expanding dark circle (Weber contrast = −0.98) on a gray background (luminance = 7.95 cd/m^2^) [4]. The standard circle subtended a visual angle of 2.6° at onset and expanded linearly at 224°/s over 200 ms to 47.4°, at which it remained for 250 ms. In Figure 3G, the expansion rate of the circle was 11.2°/s over 4 s, and the expanded size was maintained for 1,250 ms.

## Author Contributions

R.V. and T.B. designed the study and experiments. R.V. performed all experiments with assistance from D.A.E. R.V and T.B. analyzed the data. T.B. wrote the manuscript with input from R.V. and D.A.E.

## Supplemental Information

Document S1. Supplemental Experimental Procedures and Figures S1–S3

Movie S1. Examples of Mice Spontaneously Finding the Shelter during Exploration, and Fleeing to the Shelter in Response to Expanding Spots Delivered On-Path and On-Top, and to Ultrasonic Sweeps, Related to Figure 1

Movie S2. Illustrative Defensive Responses to Threat after Acute Spatial Changes in the Arena and When There Is No Arena Illumination, Related to Figure 2

Movie S3. Example of a Secondary Flight after Switching the Shelter Location and of a Freezing Response to a Slowly Expanding Spot in the Absence of Shelter, Related to Figure 3

Document S2. Article plus Supplemental Information
