# Supplementary material for: Rapid Spatial Learning Controls Instinctive Defensive Behavior in Mice
Source: Curr Biol. 2017 May 8;27(9):1342–9. doi: 10.1016/j.cub.2017.03.031 (PMC5434248; doi:10.1016/j.cub.2017.03.031)
Supplement: Document S1. Supplemental Experimental Procedures and Figures S1–S3 [file mmc1.pdf]

Current Biology, Volume *27*

# Supplemental Information

# Rapid Spatial Learning Controls

# Instinctive Defensive Behavior in Mice

**Ruben Vale, Dominic A. Evans, and Tiago Branco**

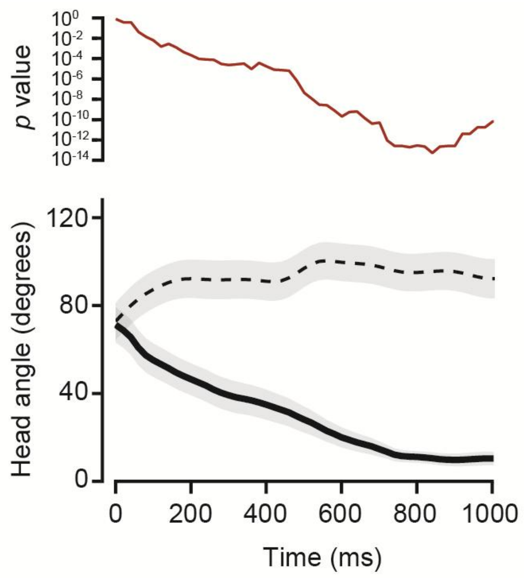


**Figure S1 – Statistical test of head rotation profiles during escape. Related to Figure 1**

Bottom, evolution over time of head angles upon threat presentation (solid line, same data shown in Figure 1G, pooled for all three different stimuli) and head angles over the same duration of time without stimulation, obtained by shuffling stimulation times across mice (dashed line). Top, *p* value for the comparison between the two angle distributions for each time point, obtained with Kolmogorov–Smirnov tests. The two distributions are significantly different after 100 ms. Shaded areas are SEM.

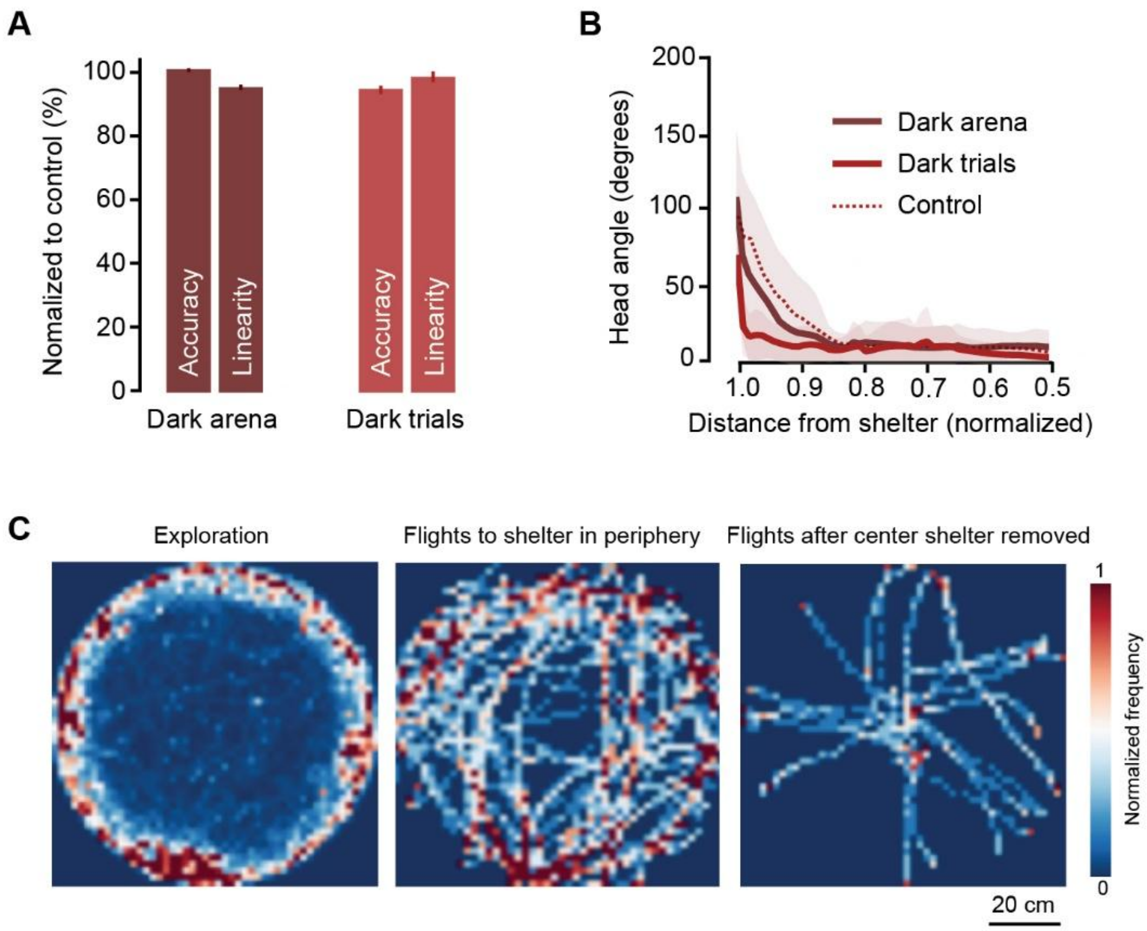


**Figure S2 – Escape behaviour in the dark and arena center aversion. Related to Figure 2**

Escape in the absence of light was tested in two different conditions: dark arena, where the arena was dark for the whole duration of the experiment, and dark trials, where the lights were turned off just before threat presentation (sound). Error bars are the SEM. (A) Accuracy and linearity are not significantly different when compared to escape responses to sound with lights on (t test against control, $P > 0.3$ for all comparisons, $n = 18$ trials from 6 animals for both conditions). (B) Head-rotation profile is similar between the two dark conditions and control, with animals correctly orienting to the shelter location before the onset of flight. (C) 2D histograms for the position of all mice during exploration (left), flights to shelters located in the arena periphery (center) and flights after the center shelter was removed (right). Unless mice have experienced a shelter in the arena center, they actively avoid the arena center during exploration (probability of stopping in the center is < 0.005) and during escape runs to the periphery (11/87 flights go through the center and for these, the probability of stopping in the center is 0.09).

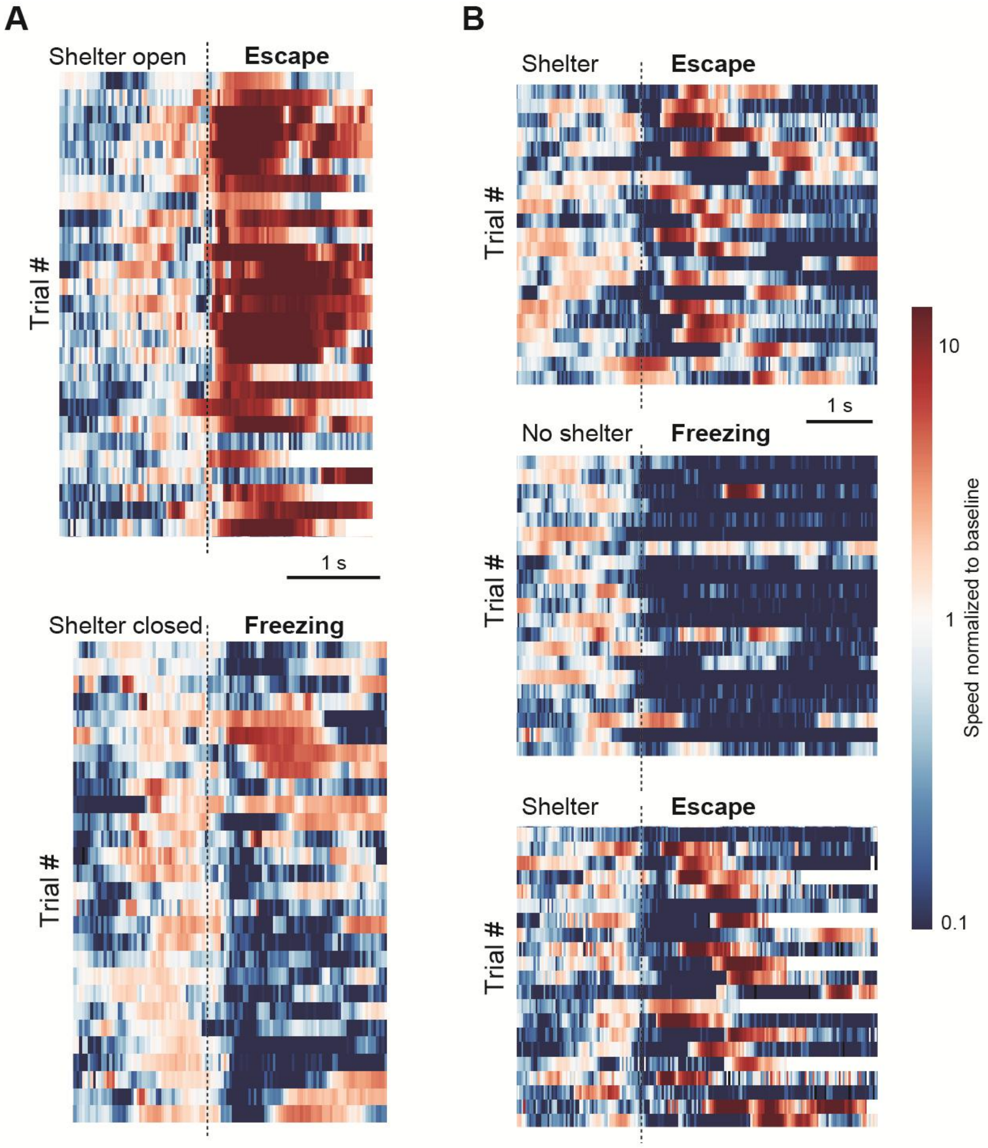


**Figure S3 – Updates of defensive strategy in different conditions. Related to Figure 3**

**(A)** Raster plots showing speed profiles upon threat stimulation before (bottom) and after the shelter hole has been opened (top), for fast expanding spots in the Barnes Maze. Trials have been aligned by reaction time (dashed line). **(B)** Raster plots for speed profiles with slowly expanding spot stimulation in a cylinder arena showing that the update of defensive strategy is reversible. Dashed line indicates onset of stimulation. Color bar applies to both panels.

## Supplemental Experimental Procedures

### *Animals*

Male C57BL/6J mice were obtained from Charles Rivers and used for experiments at 6-12 weeks old. Animals were single housed at least 72 h before experiments and maintained on a 12 h light cycle with free access to chow and water, and testing was conducted during the light phase of the light cycle. All experiments were performed under the UK Animals (Scientific Procedures) Act of 1986 (PPL 70/7652) following local ethical approval.

### *Behavioral apparatus*

The standard behavioral arena was a modified Barnes maze [S1], consisting of a white acrylic circular platform 92 cm in diameter with 20 equidistant circular holes (each 5 cm in diameter and 5.4 cm away from the border of the arena), 18 of which were permanently closed with a black plastic cover. The remaining two, which occupied opposite positions in the platform (180º), could either be closed or connected to a black Perspex shelter (dimensions: 15 x 5.8 x 4.7 cm). The central area of the arena (22 cm diameter) was a fixed circular platform, and the periphery (70 cm diameter) was mounted on a frame that allowed rotation over 360º. Rotation was implemented with a stepper motor controlled remotely. The maze was surrounded by visual cues consisting of 2D printed symbols with a variety of shapes, colors and patterns, with dimensions ranging from 100 to 310 $cm^2$ and attached to inside of the arena cabinet unless otherwise noted. Bedding from the home-cage of the mouse being tested was placed inside the shelter to serve as an olfactory cue. Illumination for video recording was provided by six infrared LED illuminators (TV6700, Abus) distributed above the arena. In addition, a DLP projector (IN3126, InFocus) was used to deliver visual stimuli (see below) and illuminated a screen above the arena with a gray background in all experiments unless otherwise noted, providing a mean illumination of 6.74 lux at the center of arena floor. The entire maze was enclosed by a black sound-deadening cabinet.

Modifications:

<u>Figure 1H</u>: A subset of experiments used an acrylic rectangular shelter (19 x 13.5 x 9.7 cm) with one entrance facing the arena center, placed peripherally, in a similar position to the maze shelter entry.

<u>Figure 2A-E</u>: The maze was surrounded by a black opaque octagonal wall (60 cm height), to block visual cues outside the apparatus. The visual cues were attached to the rotating part of the arena. Additionally, a green LED was placed on the edge of the arena in alignment with the shelter hole, and a Petri dish (35 mm) containing home-cage bedding was placed in the central area of the maze to attract the mouse and allow rotation of the arena periphery.

<u>Figure 2F-H</u>: An acrylic translucent red semi-spherical shelter, with three equidistant entries (12 cm diameter), was placed in the center of the maze, while all maze holes were covered with white disks.

<u>Figure S3B</u>: A cylindrical arena 60 cm in diameter with red acrylic walls (40 cm height) and containing a red rectangular shelter (same as in Figure 1H) was positioned under the same projector setup as described above.

***Behavioral procedures***

Experiments were recorded at 30 or 50 frames per second with a near-infrared camera (acA1300-60gmNIR, Basler) positioned above the arena. Video recording and stimulus delivery was controlled with software custom-written in LabVIEW (2015 64-bit, National Instruments), and the center of mass of the animal was tracked on-line. For all experiments, animals were placed in the arena by hand and never removed forcibly from the shelter, unless the experiment was terminated. Unless otherwise noted, animals were given a 7 min acclimation period, and an additional 5 min if they did not visit the shelter at least once. If the shelter was not found in this period, the experiment was terminated.

Figure 1A-G: Up to five stimulation trials of the same modality were delivered in a session, with a minimum interval of 60 s between stimuli.

Figure 1H-I: Trials with auditory stimulation inside and outside the shelter were interleaved, and a minimum of four and a maximum of ten responses per mouse were measured in a single session. The dataset includes five mice tested in the standard maze shelter, and six mice tested in a rectangular shelter 'above ground' (described above). The stimulation sound pressure inside the shelter was within 2 dB of the sound pressure outside.

Figure 2A-E: For each animal, three flight responses were elicited before arena rotation, and one after rotation.

Figure 2F-H: A semi-spherical shelter (described above) was placed in the arena center before introducing the mouse. After eliciting three flight responses the shelter was manually removed, and a stimulus immediately delivered (within 30 s). Additional stimuli were triggered with a minimum interval of 45 s.

Figure 3A-C: Stimulation was delivered after the animal entered the shelter for the first time, with no acclimatization period. Shelter entry was defined by the body, front and hind limbs being inside the shelter.

Figure 3D-E: After eliciting one flight, the shelter was closed while the animal was in the arena, and another shelter (180º opposite) was opened. Stimulation was delivered as soon as the animal had spontaneously entered the new shelter location once.

Figure 3F-G: Animals explored the maze with all shelters closed for 7 min, after which three stimuli were delivered. The shelter hole was then opened, and after 5 min of exploration, during which all mice found the shelter, three additional stimuli were given.

Figure S2: Experiments were run in the dark (0.01-0.04 lux) in two different conditions: 1) blacking out the arena throughout the exploration and stimulation periods (dark arena), and 2) blacking out the arena 2-4 s before the onset of each stimulation trial (dark trials).

Figure S3B: Three slowly expanding spot stimuli were delivered after 7 min of acclimation, after which the shelter was removed from the cylindrical arena and the mouse given 5 min to explore the environment again. Following three additional stimuli, the same shelter was reintroduced, and after a further 5 min of exploration, three more stimuli were delivered.

***Auditory and Visual stimulation***

The auditory stimulus consisted of a train of three frequency modulated upsweeps from 17 to 20 kHz over 3 s [S2], lasting 9 s in total. Waveforms were produced in MATLAB (Mathworks), and sound was generated by an ultrasound speaker (L60, Pettersson) positioned centrally 50-56 cm above the arena. The sound pressure measured at the arena floor was 78 dB directly underneath the speaker and 75 dB around the borders of the arena.

Visual stimuli were generated in LabVIEW and backprojected on to a 60 x 80 cm screen ('100 micron drafting film', Elmstock, UK) positioned 64 cm above the arena. The stimulus consisted of an expanding dark circle [S3], and unless otherwise stated, subtended a visual angle of 2.6º at onset and expanded linearly at 224º/s to 47.4º over 200 ms, after which it maintained the same size for 250 ms. The Weber contrast of the circle was -0.98 in all experiments, against the background luminance of the gray screen (7.95 cd/$m^2$). For the experiment shown in Figure 3G, the expansion rate of the circle was 11.2º/s over 4 s, and the expanded size was maintained for 1000 ms.

***Data analysis***

Data analysis was performed using custom-written routines in Python 2.7. Data are reported as mean ± SEM unless otherwise indicated. Statistical comparisons using the significance tests stated in the main text and figure legends were performed in SciPy Stats, and statistical significance was considered when $P < 0.05$. Flight termination in Figure 2F-H and failure to reach the correct shelter location was defined as a deceleration to less than 9 cm/s above other maze holes, and accuracy was calculated from the number of holes by which the target was missed (converted to percentage as 100% - 10% x number of holes, given that there are ten holes in 180º). The onset of stimulus-evoked escape responses was determined by visual inspection of the video recordings, and considered as the onset of head orientation to the shelter, or the onset of acceleration if the animal was already facing the shelter upon stimulation. Linearity was expressed as the percentage ratio between displacement and distance, during either the duration of the flight response or during the period between the last exit from the shelter and the subsequent sensorial stimulus (foraging linearity). Head angles were measured between 0° and 180°. For the data shown in Figure 2D-E, flights were considered as targeting either the old or new shelter location if they had an accuracy of 80-100% towards one of these targets. Animals that did not show any observable behavioral response to the sensory stimulus were excluded from the study (visual stimulus: 2/36; sound stimulus: 0/42). For the statistical testing in Figure S1, the stimulus times from all mice were shuffled and head angles measured in relation to the shelter, for the duration of the escape response corresponding to each stimulus time. If the randomly selected mouse was inside the shelter during the shuffled time, the next mouse on the randomized list was selected.

## Supplemental References

S1. Barnes, C.A. (1979). Memory deficits associated with senescence: a neurophysiological and behavioral study in the rat. Journal of Comparative and Physiological Psychology *93*, 74-104.

S2. Mongeau, R., Miller, G.A., Chiang, E., and Anderson, D.J. (2003). Neural correlates of competing fear behaviors evoked by an innately aversive stimulus. J Neurosci *23*, 3855-3868.

S3. Yilmaz, M., and Meister, M. (2013). Rapid innate defensive responses of mice to looming visual stimuli. Curr Biol *23*, 2011-2015.
