# Supplementary figures and images for: Rapid Spatial Learning Controls Instinctive Defensive Behavior in Mice
Source: Curr Biol. 2017 May 8;27(9):1342–9. doi: 10.1016/j.cub.2017.03.031 (PMC5434248; doi:10.1016/j.cub.2017.03.031)

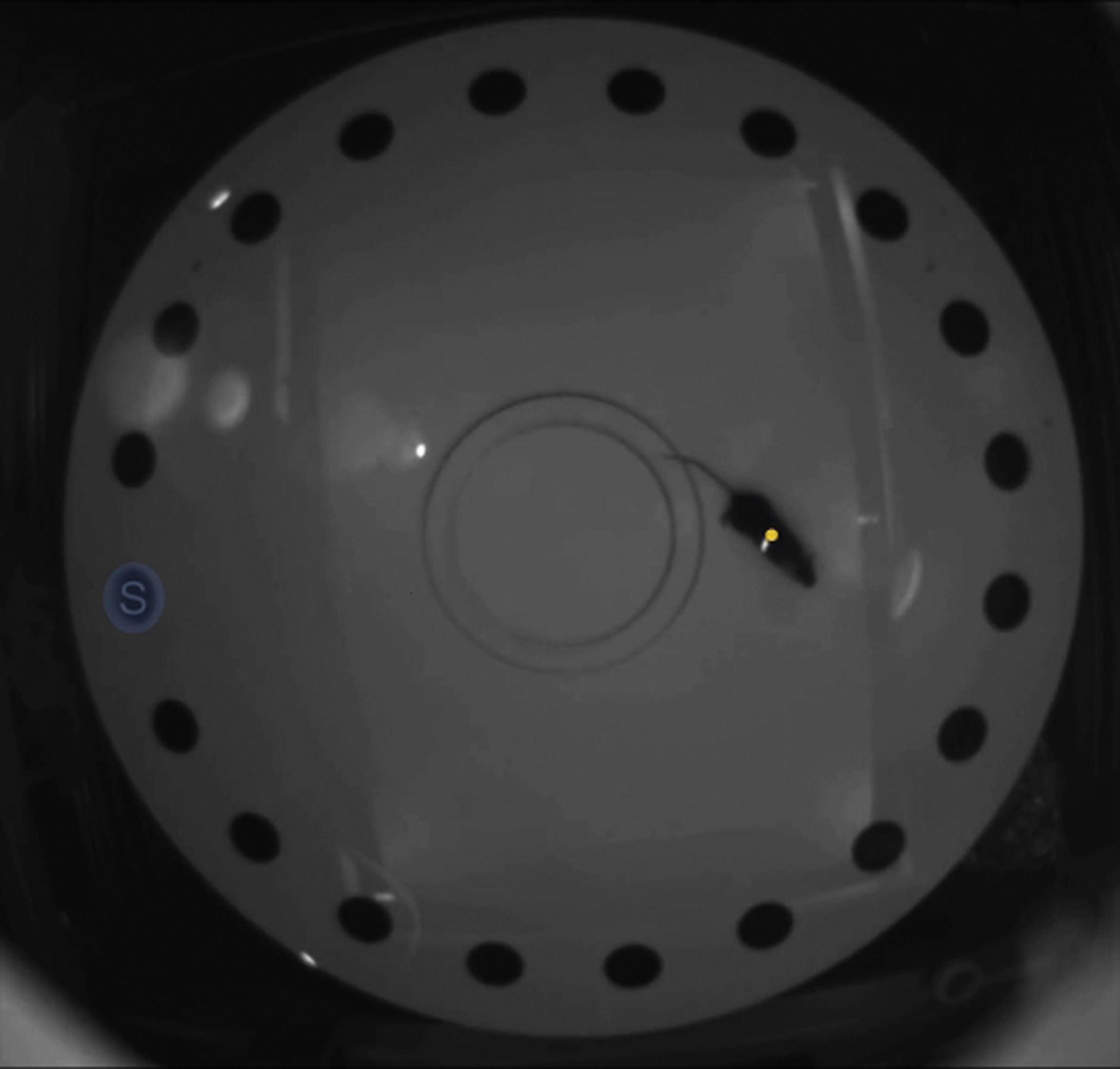

Supplement: Movie S1. Examples of Mice Spontaneously Finding the Shelter during Exploration, and Fleeing to the Shelter in Response to Expanding Spots Delivered On-Path and On-Top, and to Ultrasonic Sweeps, Related to Figure 1 [file mmc2.jpg]

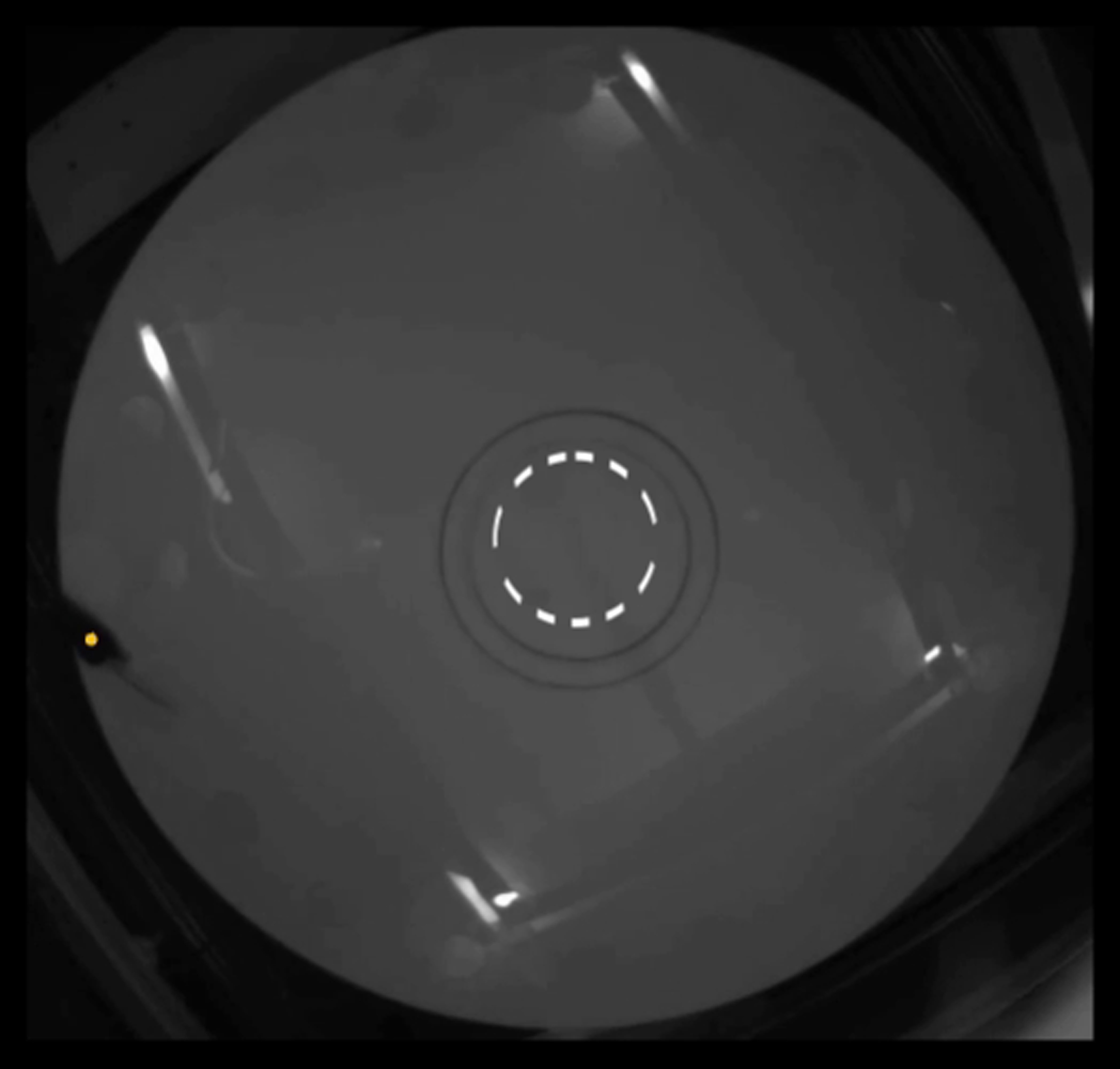

Supplement: Movie S2. Illustrative Defensive Responses to Threat after Acute Spatial Changes in the Arena and When There Is No Arena Illumination, Related to Figure 2 [file mmc3.jpg]

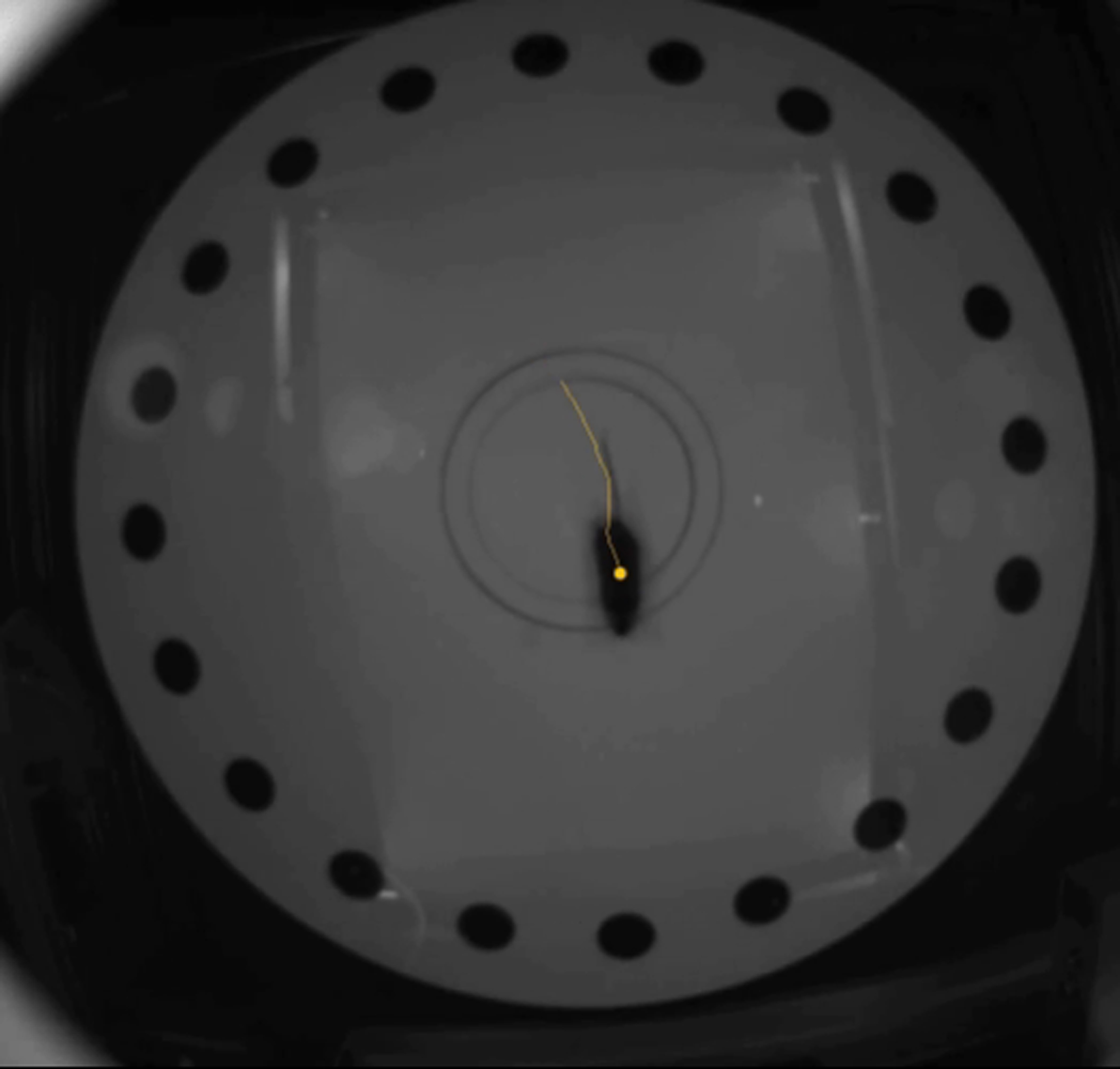

Supplement: Movie S3. Example of a Secondary Flight after Switching the Shelter Location and of a Freezing Response to a Slowly Expanding Spot in the Absence of Shelter, Related to Figure 3 [file mmc4.jpg]
